# Phenotypic Presentations of Heart Failure Among Patients With Chronic Inflammatory Diseases

**DOI:** 10.3389/fcvm.2022.784601

**Published:** 2022-03-16

**Authors:** Daniel L. Underberg, Adovich S. Rivera, Arjun Sinha, Matthew J. Feinstein

**Affiliations:** ^1^Division of Cardiology, Department of Medicine, Chicago, IL, United States; ^2^Department of Preventive Medicine, Chicago, IL, United States; ^3^Department of Pathology, Northwestern University Feinberg School of Medicine, Chicago, IL, United States

**Keywords:** heart failure, coronary artery disease, chronic inflammation, adjudication, HIV

## Abstract

**Objective:**

Characterize incident heart failure (HF) phenotypes among patients with various chronic inflammatory diseases (CIDs).

**Background:**

Several CIDs are associated with increased HF risk, but differences in HF phenotypes across CIDs are incompletely understood. No prior studies to our knowledge have manually adjudicated HF phenotypes across a CID spectrum.

**Methods:**

We screened for patients with—and controls without—CIDs who had possible HF, then hand-adjudicated HF endpoints. Possible HF resulted from a single HF administrative code; HF was deemed definite/probable vs. absent using standardized, validated criteria. We queried adjudicated HF patients' charts to define specific HF phenotypes, then compared clinical, demographic, and HF phenotypic characteristics for HF patients with specific CIDs vs. non-CID controls using Fisher's exact test.

**Results:**

Out of 415 possible HF patients, 192 had definite/probable HF. Significant differences in HF phenotypes existed across CIDs. Isolated right-sided HF was present in 27.8% of patients with SSc and adjudicated HF, which is more than twice as common as it was in any other CID. Left ventricular systolic dysfunction was most common in patients with HIV and lupus (SLE); mean LVEF was 45.0% ± 18.6% for HIV and 41.3% ± 17.1% for SLE, but was 57.7% ± 10.7% for SSc. Those with HIV and multiple CIDs were most likely to have coronary artery disease.

**Conclusions:**

Different CIDs present with different phenotypes of physician-adjudicated HF, potentially reflecting different underlying inflammatory pathophysiologies. Larger studies are needed to confirm these findings, as are mechanistic studies focused on understanding specific immunoregulatory contributors to HF.

## Introduction

Chronic inflammation plays a central role in the pathogenesis of myocardial dysfunction and heart failure (HF) (1). Yet, despite accumulating scientific evidence of the role of inflammation in HF, therapies targeting inflammation in HF treatment (2) and prevention (3) have yielded mixed results. Several reasons for this gap between scientific knowledge and practice exist, including biologic heterogeneity of heart failure phenotypes and the heterogeneity of inflammatory processes in time (even within an individual) and space (e.g., systemic vs. tissue-specific inflammation). Disentangling this heterogeneity offers the potential for useful diagnostic and therapeutic targets in HF prevention and treatment. Several human “models” of systemic and tissue-specific inflammation—chronic inflammatory diseases (CIDs) including but not limited to systemic lupus erythematosus (SLE), systemic sclerosis (SSc), and human immunodeficiency virus (HIV)—are associated with increased HF risk (4). While prior studies have yielded useful general insights into specific CIDs and incident HF, none to our knowledge have adjudicated HF subtypes and phenotypic presentations [which are essential for confirming accurate HF diagnoses given the discordance between administrative codes for HF and physician-adjudicated HF (5, 6)] to produce phenotype-specific insights. In this study, we individually adjudicated incident HF events and compared incident HF phenotypes in a large cohort of CID patients and controls.

## Methods

### Cohort Creation and Validation

Using electronic health record-based data stored in the Northwestern Medicine Enterprise Data Warehouse (NMEDW) (Northwestern University Clinical and Translational Sciences Institute, Chicago, Illinois), a large cohort of patients with CIDs and non-CID controls receiving regular outpatient care was created. Regular outpatient care was defined as at least one in-person outpatient visit every 2 years during the period of observation (1/1/2000 to 1/1/2019) in a large urban medical system. The NMEDW stores clinical observations of >6.6 million people, and its external validity is demonstrated by the fact that previous investigations of HIV-associated cardiovascular disease using this database yielded findings comparable to those of multicenter U.S. HIV cohorts (7–10). The institutional review board at Northwestern University approved the cohort creation and research protocol for this study.

We identified adults 18 years of age and older with CIDs during the above stated period of observation by using the following validated criteria: for SSc (11), inflammatory bowel disease (IBD) (12–15) and psoriasis (Pso) (16, 17), 2 or more International Classification of Diseases-9th Edition (ICD-9) or ICD-10 diagnostic codes were required within a 2-year period; for SLE, 3 diagnostic codes were required in 3 separate months (18, 19); for RA, 2 diagnostic codes and a prescription for a disease-modifying antirheumatic drug were required (20–22). To diagnose HIV, we adapted a previous approach (23) that factored in plasma HIV RNA (viral load), serology, and/or at least 3 instances in which both HIV viral load and CD4 T lymphocyte cell count (CD4) were ordered on the same date. We frequency matched non-CID controls with patients with CIDs based on several demographic factors (age, sex, insurance status) as well as baseline year and the presence or absence of hypertension and/or diabetes at baseline.

We obtained demographic and insurance data from patients' most recent clinical visits. We used an evidence-based approach to define baseline hypertension as the presence of 1 administrative code (ICD-9 401 to 405 and ICD-10 I10 to I15) anytime prior to 1 year after the baseline date (24, 25). Given the heterogeneity of practice settings and potential for sphygmomanometer miscalibration that has been demonstrated in prior studies (26, 27), blood pressure values themselves were not used to define hypertension. We identified baseline diabetes using a combination of validated ICD-9 or ICD-10 administrative codes (ICD-9 250 and ICD-10 E10-, E11, and E13) and either hemoglobin *A*_1c_ >6.5% or prescription of antidiabetic medications anytime prior to 1 year after the baseline date (28). The presence of coronary artery disease (CAD) was determined by manual chart review, and was defined as present if it was mentioned in at least 1 note written by either an Internal Medicine or Cardiology specialist in the patient's electronic health record.

### Adjudication

Our adjudication protocol was based on a method we previously adapted from the Multi-Ethnic Study of Atherosclerosis for adjudicating events in electronic health records-based cohorts (29). As incident HF was the primary outcome of interest in this study, we used intentionally sensitive screening criteria in our CID and non-CID cohorts to define this; patients were deemed to have “possible heart failure” based on a single inpatient or outpatient administrative code (ICD-9 codes 398.91, 402.01, 402.11, 402.91, 404.01, 404.03, 404.11, 404.13, 404.91, 404.93, 425, 428, 429 or ICD-10 codes I42, I43, or I50). Only HF events coded >1 year after baseline were consideredw incident diagnoses in order to minimize the likelihood of misclassifying prevalent but not yet recorded HF as incident HF; importantly, HF events coded within the first year after baseline were considered baseline. Patients deemed to have HF at baseline and those with missing baseline demographic data were excluded.

Patients who screened positive for “possible heart failure” subsequently underwent manual chart review to determine whether “probable heart failure” was present at the time of incident HF diagnosis. This designation was based on fulfilling all 3 of the following criteria: (1) the patient had signs or symptoms of HF (shortness of breath, lower extremity swelling, or jugular venous distension not attributable to a clear other cause); (2) the physician diagnosed HF (by mentioning “heart failure” or “volume/fluid overload” in the setting of objective criteria on echocardiogram [EF <55% or ≥ grade II diastolic diastolic dysfunction] or other superseding diagnoses including “severe valvular disease” or other “cardiomyopathy”); and (3) the patient was prescribed medication for HF (beta-blockers, angiotensin II converting enzyme inhibitors, angiotensin receptor blockers, mineralocorticoid antagonists, angiotensin receptor-neprilysin inhibitors, or diuretics).

Patients screening positive for “probable heart failure” underwent further manual chart review to determine whether “definite heart failure” was present at the time of incident HF diagnosis. This designation was made by fulfilling at least 1 of the following criteria: (1) chest x-ray showed signs of volume overload; (2) echocardiogram showed evidence of HF; or (3) BNP or NT-proBNP was consistent with HF (using age-adjusted cutoff values of ≥450 for age <50, ≥900 for age 50–75, and ≥1,800 for age >75). Echocardiographic evidence of HF was defined as meeting at least 1 of the following criteria, based on the lowest values seen on echocardiogram within 1 month of incident HF diagnosis: (1) left ventricular ejection fraction <50; (2) wall motion abnormalities; (3) left ventricular systolic dysfunction (chamber dilation or left ventricular end diastolic volume index >97 ml/m^2^); (4) diastolic dysfunction (left ventricular end diastolic pressure >16mmHg, pulmonary capillary wedge pressure >12mmHg, E/E' >15, or left atrial volume index >34ml/m^2^); (5) moderate-severe valvular disease; or (6) evidence of right ventricular failure (depressed systolic function, dilation, or evidence of pressure/volume overload). Definite and probable HF events were combined and considered HF events for the purpose of these analyses. In addition to determining whether HF was present or absent, we adjudicated phenotype-related factors, such as the presence or absence of CAD (as previously specified) and right vs. left-sided HF (based on echocardiographic parameters), as well as whether or not the patient received a cardiac MRI.

### Statistical Analyses

Our analyses focused on phenotypic differences *among* individuals with HF, which we determined by physician adjudication, rather than factors associated with incident HF. Therefore, our entire sub-cohort analyzed had adjudicated HF; our central analyses were performed without additional adjustment for HF risk factors because all individuals had HF, and because the stratified, CID-specific nature of the analyses limited our power for additional stratification or adjustment. Nevertheless, key demographic and clinical covariates were reviewed to assist with interpretation; these are provided along with the central analyses in Table 1 and are discussed in the results. The co-primary endpoints, compared across CIDs and vs. non-CID controls, were percentage (%) of individuals within a particular CID group with each of the following phenotypic presentations of HF: Isolated (1) left- or (2) right-sided HF, (3) combined left- and right-sided HF, and (4) left ventricular ejection fraction on echocardiography. For each of these variables, we performed analysis of variance (ANOVA) across CID/non-CID control groups with subsequent *post-hoc* Fisher's exact tests due to limited sample size for each CID/HF sub-group.

**Table 1:** Heart failure demographics, phenotypes, risk factors, and workup among patients with and without chronic inflammatory diseases who adjudicated positive for heart failure.

		**No CID**	**HIV**	**IBD**	**Pso**	**RA**	**Scl**	**SLE**	**Multiple CID**	***p*-value**
Demographics	# adjudicated (+) for HF	81	19	9	16	24	18	14	11	–
	Age at baseline [mean (SD)]	66.6 (13.4)	51.0 (9.70)	59.6 (19.0)	64.1 (12.0)	67.5 (10.6)	54.7 (11.6)	45.1 (16.6)	58.8 (9.6)	<0.001
	Male sex (%)	48 (59.3)	14 (73.7)	5 (55.6)	5 (31.3)	3 (12.5)	3 (16.7)	4 (28.6)	4 (36.4)	<0.001
	Asian/Other (%)	6 (7.4)	1 (5.3)	0	1 (6.3)	1 (4.2)	4 (22.2)	2 (14.3)	1 (9.1)	–
	Black non-hispanic (%)	19 (23.5)	12 (63.2)	1 (11.1)	1 (6.2)	6 (25)	3 (16.7)	7 (50)	3 (27.3)	–
	Hispanic (%)	3 (3.7)	1 (5.3)	0	1 (6.2)	3 (12.5)	4 (22.2)	3 (21.4)	1 (9.1)	–
	White non-hispanic (%)	53 (65.4)	5 (26.3)	8 (88.9)	13 (81.2)	14 (58.3)	7 (38.9)	2 (14.3)	6 (54.5)	–
HF phenotypes	Isolated L-sided HF (%)^*^	34 (43.0)	10 (52.6)	2 (22.2)	7 (46.7)	11 (45.8)	4 (22.2)	5 (35.7)	5 (45.5)	0.573
	Isolated R-sided HF (%)^*^	3 (3.8)	0	1 (11.1)	0	1 (4.2)	5 (27.8)	0	0	0.021
	Combined L/R HF (%)^*^	25 (31.6)	4 (21.1)	3 (33.3)	6 (40.0)	5 (20.8)	3 (16.7)	8 (57.1)	4 (36.4)	0.265
	LV systolic dysfunction^*^	38 (53.5)	10 (62.5)	4 (44.4)	5 (35.7)	6 (35.3)	1 (6.7)	11 (78.6)	2 (20.0)	0.001
	Moderate-severe valvular dysfunction^*^	27 (38.0)	4 (25.0)	4 (44.4)	2 (14.3)	2 (11.8)	5 (31.2)	4 (28.6)	3 (30.0)	0.379
	WMA (%)^*^	31 (44.3)	9 (56.2)	4 (44.4)	6 (42.9)	5 (31.2)	2 (13.3)	9 (64.3)	2 (20.0)	0.094
	BNP c/w HF (%)	12 (19.7)	10 (55.6)	5 (62.5)	4 (33.3)	4 (25.0)	4 (25.0)	10 (71.4)	2 (25.0)	0.002
	Echo LVEF [mean (SD)]^*^	47.16 (15.0)	44.97 (18.6)	46.33 (22.3)	53.85 (17.9)	51.18 (17.9)	57.66 (10.7)	41.29 (17.1)	59.56 (12.5)	0.038
HF risk factors	CAD (%)	45 (57.0)	13 (68.4)	3 (33.3)	5 (33.3)	12 (50.0)	6 (33.3)	6 (42.9)	6 (60.0)	0.226
	HTN (%)	15 (18.5)	11 (57.9)	0	6 (37.5)	5 (20.8)	6 (33.3)	3 (21.4)	3 (27.3)	0.013
	DM (%)	10 (12.3)	9 (47.4)	1 (11.1)	5 (31.2)	5 (20.8)	0	3 (21.4)	3 (27.3)	0.008
	A-fib (%)	24 (29.6)	4 (21.1)	4 (44.4)	4 (25.0)	5 (20.8)	5 (27.8)	2 (14.3)	2 (18.2)	0.768
	COPD (%)	16 (19.8)	3 (15.8)	0	3 (18.8)	1 (4.2)	2 (11.1)	1 (7.1)	1 (9.1)	0.446
	Used DMARD (%)	10 (12.3)	0	3 (33.3)	9 (56.2)	23 (95.8)	8 (44.4)	11 (78.6)	9 (81.8)	<0.001
	Used gluco-Corticoids (%)	14 (17.3)	1 (5.3)	1 (11.1)	7 (43.8)	16 (66.7)	5 (27.8)	9 (64.3)	6 (54.5)	<0.001
HF workup	Got CMR	17 (21.5)	6 (31.6)	3 (33.3)	4 (26.7)	7 (29.2)	9 (50.0)	6 (42.9)	2 (20.0)	0.339

* For ehocardiographic measurements indicated with an asterisk (^*^), there were an average of 90.8% with no CID, 91.0% with HIV, 100% with IBD, 89.3% with Pso, 82.7% with RA, 92.1% with Scl, 100.0% with SLE, and 89.6% with multiple CIDs who had complete echo measurements. Percentages of variables marked with (^*^) were calculated using a denominator of those with complete echo data.

*CID, chronic inflammatory disease; HIV, human immunodeficiency virus; IBD, inflammatory bowel disease; Pso, psoriasis; RA, rheumatoid arthritis; Scl, scleroderma; SLE, systemic lupus erythematosus*.

## Results

Out of 415 patients with CIDs and controls with at least one administrative code for HF, 192 had definite/probable HF, consistent with expected high false positivity screening rates of our intentionally sensitive approach to screening for possible HF events. We observed significant differences in HF phenotypes across CIDs (Table 1). Isolated right-sided HF was present in 27.8% of patients with SSc and adjudicated HF, which is more than twice as common as it was in any other CID. Left ventricular systolic dysfunction was most common in patients with HIV and SLE; mean LVEF was 45.0 ± 18.6% for HIV and 41.3 ± 17.1% for SLE, whereas it was 57.7 ± 10.7% for SSc. CAD was most prevalent in those with adjudicated HF who had HIV (68.4% of patients with HIV and HF had CAD) and multiple CIDs (60% of patients with multiple CIDs had CAD). Hypertension and diabetes were also most common in adjudicated HF patients with HIV (present in 57.9 and 47.4% of patients with HIV, respectively). We did not observe significant differences in the prevalence of isolated left-sided HF or atrial fibrillation between the various CIDs.

In a secondary analysis comparing proportions of each CID group that had HF with preserved ejection fraction (HFpEF) as the presentation of HF, SSc (in which 66.7% of HF cases were adjudicated as HFpEF) and Pso (60.0%) had the highest proportion of HFpEF, whereas only 38.0% of non-CID controls with HF had HFpEF.

## Discussion

We adjudicated HF events in a large cohort of CID patients and controls and identified significant differences in adjudicated HF subtypes across CIDs. This represents the first study to our knowledge that individually adjudicated HF events and phenotypes in a cohort examining patients with a spectrum of CIDs.

We observed that isolated right-sided HF was much more common in SSc than in any other CID, and that left ventricular systolic dysfunction was more common in HIV and SLE. There was no significant difference in the rate of isolated left-sided HF across the spectrum of CIDs, however. Our findings substantiate previously postulated (4, 30, 31) CID-specific HF phenotypes, with our study adding the value of enabling comparison across CIDs in a single cohort and presenting physician-adjudicated assessment and phenotyping of HF, which may be more accurate than administrative code-based definitions (32). Additionally, although pulmonary hypertension—a key contributor to right-sided HF—is common in several CIDs including SSc, HIV, and SLE, we observed a stark difference whereby SSc patients with HF had a several-fold higher likelihood of isolated right-sided HF than patients with any other CID and HF. Whether these differences reflect unique heart-lung physiology and fibrotic mediators arising in SSc warrants further study. Moreover, although several CIDs (including HIV, RA, and SLE) are associated with heightened atherothrombotic risk, HF patients with HIV had an especially high prevalence of underlying CAD; this adds to recently reported data revealing a higher rate of CAD in patients with SLE and SSc (33), and substantiates prior reports highlighting HIV's association with acute coronary syndrome (34).

Although these findings yield interesting CID-specific insights into HF presentation and, perhaps, underlying pathophysiologies, they require confirmation in larger, multi-center studies as one of the limitations of our current work is the relatively small sample size. Such analyses would offer the benefit of providing CID-specific insights into HF risk and pathophysiology.

## Data Availability Statement

The raw data supporting the conclusions of this article will be made available by the authors, without undue reservation.

## Ethics Statement

The studies involving human participants were reviewed and approved by Northwestern University IRB. Written informed consent for participation was not required for this study in accordance with the national legislation and the institutional requirements.

## Author Contributions

DU, AS, and MF constituted the team that conceptualized the project, collected and reviewed the data, and wrote and edited the final manuscript. AR assisted with statistical analyses. All authors contributed to the article and approved the submitted version.

## Funding

This study was supported by the National Institutes of Health R01 HL 156592 (to MF).

## Conflict of Interest

MF was employed by Consultant/Advisory Board, Novartis AG. The remaining authors declare that the research was conducted in the absence of any commercial or financial relationships that could be construed as a potential conflict of interest.

## Publisher's Note

All claims expressed in this article are solely those of the authors and do not necessarily represent those of their affiliated organizations, or those of the publisher, the editors and the reviewers. Any product that may be evaluated in this article, or claim that may be made by its manufacturer, is not guaranteed or endorsed by the publisher.
